# Twenty-five-year mapping species of the superfamily Cercopoidea (Hemiptera, Auchenorrhyncha) in Bulgaria

**DOI:** 10.3897/BDJ.12.e124720

**Published:** 2024-06-04

**Authors:** Radost Angelova, Ilia Gjonov

**Affiliations:** 1 Sofia University, Faculty of Biology, Sofia, Bulgaria Sofia University, Faculty of Biology Sofia Bulgaria

**Keywords:** Aphrophoridae, Cercopidae, biogeography, the Balkans, occurrence dataset

## Abstract

**Background:**

In Bulgaria, the superfamily Cercopoidea consists of 18 species in two families - Aphrophoridae and Cercopidae. Of these, 13 species of Aphrophoridae belong to the genera *Philaenus*, *Neophilaenus*, *Aphrophora* and *Lepyronia* and five species of Cercopidae are in *Cercopis* and *Haematoloma*. Over a period of 25 years of extensive research on the species of the superfamily in the country, a large amount of geo-referenced data has been collected on 17 of the species, which has significantly increased knowledge of their biogeography.

**New information:**

The paper presents a dataset of the materials of the superfamily Cercopoidea deposited in the Zoological Collection of the University of Sofia (BFUS). The specimens were collected from 888 localities in Bulgaria over a period of 25 years (1997 to 2022). The Cercopoidea collection comprises 8722 specimens grouped into 6670 collection objects.

The text provides data for each species, including a distribution map, regional literature taxon names and identifiers from eight taxonomic infrastructures (GBIF, BOLD, OpenBiodiv, BHL, COL, Plazi, EOL and TaxonWorks). It also includes data from literature and new records, phenology and altitudinal distribution in Bulgaria, as well as known host plants. Live photographs are provided for all species. A nanopublication presents the establishment of a new host plant, *Asphodelinelutea* (L.) Rchb., for the species *Philaenussignatus* Melichar, 1896.

## Introduction

The superfamily Cercopoidea (Hemiptera, Auchenorrhyncha), commonly known as spittlebugs, feed on xylem sap from a variety of plant hosts. Although some are monophagous throughout their lives or only in the nymphal stage, others are known to be very broadly polyphagous as *Philaenusspumarius* (L.) is known to be the most polyphagous phytophage (Thompson et al. 2023). Their colloquial name derives from the distinctive developmental pattern in their nymphal stages, which is characterised by the production of froth or "spittle", presumably as a protective covering. This foam, which is unique to insect nymphs, is produced when nymphs expel excess fluid mixed with products from the Malpighian tubes and possibly the abdominal epidermal Batelli glands through their anal opening while drawing in air, resulting in a foamy emulsion (Weaver and King 1954, Rakitov 2002). During the entire pre-imaginal stage, the froth maintains a moist consistency, resembling spittle deposited upon the foliage (Weaver and King 1954).

Globally, the superfamily Cercopoidea consists of six extant families: Aphrophoridae Amyot & Serville, 1843, Cercopidae Leach, 1815, Clastopteridae Dohrn, 1859, Epipygidae Hamilton, 2001, Machaerotidae Stål, 1866 and Ischnorhinidae Schmidt, 1920. These families comprise 2936 recognised species in 374 genera, with new taxa still being discovered (Soulier-Perkins 2019, Dmitriev et al. 2022, Crispolon et al. 2023).

Bulgaria has a documented diversity of 17 species within this superfamily (Nedjalkov 1908, Joakimov 1909, Lindberg 1949, Dirimanov and Harizanov 1964, Cantoreanu and Gruev 1967, Pelov 1968, Bajrjamova 1970, Bajrjamova 1972, Bayryamova 1976, Bairamova 1978, Bajrjamova 1984, Bajrjamova 1988, Bajrjamova 1990, Gjonov 2004, Angelova and Gjonov 2022). Notably, Aphrophoridae is represented by four genera (*Philaenus*, *Neophilaenus*, *Aphrophora* and *Lepyronia*) and 12 species, while Cercopidae includes two genera (*Cercopis* and *Haematoloma*) and five species. However, the presence of *Haematolomadorsatum* (Ahrens, 1812) in Bulgaria is uncertain, as it has only been mentioned in Nast (1987) without a specific location.

The objective of this study is to present the results of a 25-year (1997 to 2022) sampling of Cercopoidea species in Bulgaria. The data presented in this study serve as a comprehensive reference for future research and monitoring. The dataset is published in GBIF and is open access, compliant with FAIR principles.

## Materials and methods

The materials used in this study consist of 8722 specimens, which are organised into 6670 collection objects, each with a unique identification number. These specimens were collected from 888 localities in Bulgaria between 1997 and 2022 and are deposited in the Zoological Collection of the University of Sofia (BFUS). They were sampled by the authors in a variety of locations across the country, with varying levels of intensity and effort. Some localities were visited on multiple occasions.

The specimens were collected using a sweeping net and subsequently extracted with an aspirator. Ethyl acetate vapour was used to kill the specimens. To preserve them for long-term storage, they were placed on layers of cotton. The majority of the specimens were pinned on to glue boards. In cases where there were too many, they were separated by sex, counted and placed in entomological pin-mounted gelatin capsules and polypropylene tubes. The genital apparatus of the male specimens was dissected to observe their identifying characteristics. The material was identified by referring to the latest literature (Drosopoulos and Asche 2000, Drosopoulos and Remane 2000, Drosopoulos 2003, Biedermann and Niedringhaus 2009) and databased using Specify 7 Collection software. The software generated three labels for each collection item: 1. collection event data; 2. determination; 3. individual collection object number and QR code. The labels were cut using a cutting plotter, mounted on to the pins of the collection objects by using of a 3D pinning block (Gjonov 2022, Gjonov and Hristozov 2024). Distribution maps were created using the free and open source programme QGIS 3.28 (http://www.qgis.org) and MapTiler shapefile (https://www.maptiler.com/).

The species accounts include distribution maps, identifiers from eight taxonomic infrastructures (GBIF, BOLD, OpenBiodiv, BHL, COL, Plazi, EOL and TaxonWorks), regional literature taxon names (chresonymy), as well as literature and new records, phenology, altitudinal distribution in Bulgaria and known host plants. Live photographs are also included for all species. Unless otherwise indicated, the images presented are of specimens collected from Bulgaria. For some species, additional notes on habitat and frequency of occurrence are included.

The finding of a new host plant for *Philaenussignatus* Melichar, 1896 was presented as a nanopublication.

A dataset with all the occurrences was created and published in GBIF portal under CC-BY licence Angelova and Gjonov (2024), https://doi.org/10.15468/hc7eyy.

## Checklists

### Superfamily Cercopoidea Leach, 1815

#### Aphrophoridae

Amyot & Serville, 1843

71E76904-3942-5666-9FD0-943B1DDF83E0

#### Aphrophora

Germar, 1821

42332621-C736-5B56-AC10-2E6131ADFD98

#### Aphrophora alni

(Fallén, 1805)

FD9971CF-36EB-5DFE-821F-A99967B56FE3

https://www.gbif.org/species/2016266

https://www.boldsystems.org/index.php/TaxBrowser_TaxonPage?taxid=198947

https://openbiodiv.net/a6f5ae7c-c7f5-4f62-b557-26c4a9434201

https://www.biodiversitylibrary.org/namedetail/Aphrophora_alni_(Fall%C3%A9n%2C_1805)

https://www.catalogueoflife.org/data/taxon/FKF2

https://treatment.plazi.org/id/03BD1B1BFFABFFFE4DF0FF778B40EE35

https://eol.org/pages/2865796

46e478cb-7e0c-4372-a2ac-d12478225a87

##### Notes

Distribution map (Fig. 1a) and habitus (Fig. 1b).

**Literature data**: Northern and eastern Bulgaria (Nedjalkov 1908); Pre-Balkan (Joakimov 1909, Lindberg 1949, Bairamova 1978)⁠; Western Stara Planina Mts. (Bairamova 1978); Central Stara Planina Mts. (Cantoreanu and Gruev 1967); Vitosha Mts. (Joakimov 1909); Sofia Plain (Bajrjamova 1970, Bairamova 1978); Upper Thracian Lowland (Dirimanov and Harizanov 1964)⁠; ⁠Krupnik-Sandanski-Petrich Valley (Sander 1985)⁠; Belasitsa Mts. (Bajrjamova 1988)⁠; Rila Mts. (Lindberg 1949, Sander 1985); Rhodopes (Cantoreanu and Gruev 1967, Sander 1985, Gjonov 2004); Strandzha Mts. (Bairamova 1978); Southern Black Sea coast (Joakimov 1909, Cantoreanu and Gruev 1967, Bayryamova 1976, Bairamova 1978, Sander 1985)⁠.

**New data**: Danube Plain; Dobrudzha; Northern Black Sea Coast; Pre-Balkan; Western, Central and Eastern Stara Planina Mts.; Kraishtensko-Konjavo Region; Sofia Plain; Sub-Balkan valleys; Vitosha Mts.; Lozenska Planina Mts.; Sredna Gora Mts.; Ograzhden Mts.; Belasitsa Mts.; ⁠Krupnik-Sandanski-Petrich Valley; Rila Mts.; Pirin Mts.; Mesta River Valley; Rhodopes; Slavjanka Mts.; Upper Thracian Lowland; Strandzha Mts.; Southern Black Sea coast. Detailed occurrence data: Angelova and Gjonov (2024).

**Phenology**: April-November.

**Altitudinal distribution**: 0-2100 m a.s.l.

**Host plant**: Polyphagous (Nickel 2003).

**Additional notes**: Common in Bulgaria.

#### Aphrophora corticea

Germar, 1821

C2A9C3C1-2791-5F81-A3C8-A5FD1AAD4305

https://www.gbif.org/species/2016232

http://www.boldsystems.org/index.php/TaxBrowser_TaxonPage?taxid=674325

https://openbiodiv.net/63cb3ec4-0149-45fa-8daf-4faf3e409551

https://www.biodiversitylibrary.org/namedetail/Aphrophora_corticea

https://www.catalogueoflife.org/data/taxon/FKG7

Not available

https://eol.org/pages/3741066

614c88e1-c196-47d5-9216-fe2aafdff7fc

##### Notes

Distribution map (Fig. 2a) and habitus (Fig. 2b).

**Literature data**: Vitosha Mts. (Bajrjamova 1972).

**New data**: Kraishtensko-Konjavo Region; Vitosha Mts.; Lozenska Planina Mts.; Rhodopes. Detailed occurrence data: Angelova and Gjonov (2024).

**Phenology**: July-October.

**Altitudinal distribution**: 650-1850 m a.s.l.

**Host plant**: Nymphs on various plants in the understorey of pine saplings, adults on *Pinussylvestris* L. (Nickel 2003).

**Additional notes**: Rare in Bulgaria.

#### Aphrophora salicina

(Goeze, 1778)

32F5A77A-EC5C-5503-BA8A-9A945076FD67

https://www.gbif.org/species/4482772

http://www.boldsystems.org/index.php/Taxbrowser_Taxonpage?taxid=455312

https://openbiodiv.net/9284b773-b609-4bd1-ac8d-70a8415f88e0

https://www.biodiversitylibrary.org/namedetail/Aphrophora_salicina

https://www.catalogueoflife.org/data/taxon/FKKP

https://treatment.plazi.org/id/03BD1B1BFFABFFFE4DF0FA5F8CE8EC00

https://eol.org/pages/3741062

0842b070-a8e8-4d9f-aa40-cff8dd9c96cf

##### Notes

Distribution map (Fig. 3a) and habitus (Fig. 3b).

**Chresonymy**: *Aphrophorasalicis* Degeer in Nedjalkov (1908); *Aphrophorasalicis* de Geer in Joakimov (1909); *Aphrophorasalicis* De Geer, 1773 in Dlabola (1957); *Aphrophorasalicis* De Geer in Pelov (1968); *Aphrophoracostalis* Matsmura in Sander (1985); *Aphrophoracostalis* Mats. in Nast (1987); *Aphrophorapectoralis* (Matsumura, 1903) in Angelova and Gjonov (2022)

**Literature data**: Northern and eastern Bulgaria (Nedjalkov 1908); Danube Valley (Pelov 1968); Sofia Plain (Joakimov 1909, Pelov 1968, Sander 1985); Sredna Gora Mts. (Joakimov 1909, Angelova and Gjonov 2022); Krupnik-Sandanski-Petrich Valley (Sander 1985); Rhodopes (Joakimov 1909, Gjonov 2004); Sakar-Tundzhan Region (Dlabola 1957).

**New data**: Danube Plain; Dobrudzha; Northern Black Sea Coast; Central and Eastern Stara Planina Mts.; Kraishtensko-Konjavo Region; Sofia Plain; Sub-Balkan valleys; Vitosha Mts.; Sredna Gora Mts.; Krupnik-Sandanski-Petrich Valley; Rila Mts.; Mesta River Valley; Rhodopes; Slavjanka Mts.; Upper Thracian Lowland, Strandzha Mts.; Southern Black Sea coast. Detailed occurrence data: Angelova and Gjonov (2024).

**Phenology**: May-September.

**Altitudinal distribution**: 5-1600 m a.s.l.

**Host plant**: Olygophagous on *Salix* sp. (Nickel 2003).

**Additional notes**: Common in river valleys.

#### Lepyronia

Amyot & Serville, 1843

19BA75D3-EFC8-575C-853C-A7E825626B43

#### Lepyronia coleoptrata

(Linnaeus, 1758)

B2D9034C-CB4B-5979-B01C-4A0246955FEB

https://www.gbif.org/species/2015789

http://www.boldsystems.org/index.php/Taxbrowser_Taxonpage?taxid=198949

https://openbiodiv.net/ed0ee523-6500-4d9a-8271-3d4149563b2c

https://www.biodiversitylibrary.org/namedetail/Lepyronia_coleoptrata

https://www.catalogueoflife.org/data/taxon/6Q22Q

https://treatment.plazi.org/id/03BD1B1BFFAAFFF94DF0F9CD8D7EED98

https://eol.org/pages/627808

ca1a1a8d-dadb-451e-80d8-b31dc25b10d7

##### Notes

Distribution map (Fig. 4a) and habitus (Fig. 4b).

**Literature data**: Northern and eastern Bulgaria (Nedjalkov 1908); Danube Plain (Angelova et al. 2008); Vitosha Mts. (Lindberg 1949)⁠⁠⁠⁠⁠⁠⁠⁠⁠⁠⁠⁠⁠⁠⁠⁠⁠⁠⁠⁠; Sofia Plain (Bajrjamova 1988)⁠⁠⁠⁠⁠⁠⁠⁠⁠⁠⁠⁠⁠⁠⁠⁠; Lozen Mts. (Bayryamova 1976)⁠; Pre-Balkan (Joakimov 1909, Lindberg 1949)⁠⁠⁠⁠⁠⁠⁠⁠⁠⁠⁠⁠⁠⁠⁠⁠⁠⁠⁠⁠⁠⁠⁠⁠⁠⁠⁠⁠⁠⁠⁠⁠⁠⁠; Krupnik-Sandanski-Petrich Valley (Sander 1985); Belasitsa Mts. (Bajrjamova 1988); Rhodopes (Sander 1985, Gjonov 2004)⁠; Thracian Lowland (Dirimanov and Harizanov 1964, Cantoreanu and Gruev 1967); Sakar-Tundzhan Region (Joakimov 1909, Dlabola 1957)⁠.

**New data**: Danube Plain; Dobrudzha; Northern Black Sea Coast; Pre-Balkan; Western, Central and Eastern Stara Planina Mts.; Kraishtensko-Konjavo Region; Sofia Plain; Sub-Balkan valleys; Vitosha Mts.; Lozenska Planina Mts.; Sredna Gora Mts.; Ograzhden Mts.; Belasitsa Mts; Krupnik-Sandanski-Petrich Valley; Rila Mts.; Pirin Mts.; Slavjanka Mts.; Mesta River Valley; Rhodopes; Upper Thracian Lowland; Sakar-Tundzhan Region; Strandzha Mts.; Southern Black Sea coast. Detailed occurrence data: Angelova and Gjonov (2024).

**Phenology**: May-November.

**Altitudinal distribution**: 0-2100 m a.s.l.

**Host plant**: Polyphagous (Nickel 2003).

**Additional notes**: Common species.

#### Neophilaenus

Haupt, 1935

67CB8360-47FF-5449-874A-B69A56BEEAB2

#### Neophilaenus albipennis

(Fabricius, 1798)

DEBC6ED1-8B11-5176-9037-D4598B8E4200

https://www.gbif.org/species/2016796

http://www.boldsystems.org/index.php/Taxbrowser_Taxonpage?taxid=759703

https://openbiodiv.net/14f77e9d-0271-45a1-bcbb-7c282e3ca820

https://www.biodiversitylibrary.org/namedetail/Neophilaenus_albipennis

https://www.catalogueoflife.org/data/taxon/46Q4F

Not available

https://eol.org/pages/4215245

fc2720a8-b44b-4d55-94fe-2dd6d51e2004

##### Notes

Distribution map (Fig. 5a) and habitus (Fig. 5b).

**Chresonymy**: *Ptyelusalbipennis* Fabr. in Joakimov (1909)

**Literature data**: Eastern Stara Planina Mts. (Pelov 1968); Vitosha Mts. (Joakimov 1909); Krupnik-Sandanski-Petrich Valley (Sander 1985)⁠.

**New data**: Pre-Balkan; Western, Central and Eastern Stara Planina Mts.; Kraishtensko-Konjavo Region; Sofia Plain; Vitosha Mts.; Lozenska Planina Mts.; Ograzhden Mts.; Belasitsa Mts; Pirin Mts.; Slavjanka Mts.; Mesta River Valley; Rhodopes; Sakar-Tundzha Region; Strandzha Mts.; Southern Black Sea coast. Detailed occurrence data: Angelova and Gjonov (2024).

**Phenology**: May-September.

**Altitudinal distribution**: 0-2200 m a.s.l.

**Host plant**: Monophagous on *Brachypodiumpinnatum* (L.) P. Beauv. (Nickel 2003).

#### Neophilaenus campestris

(Fallén, 1805)

E3194124-FDAD-5FC5-AD0D-A2B47DA8FF78

https://www.gbif.org/species/4482781

http://www.boldsystems.org/index.php/Taxbrowser_Taxonpage?taxid=725843

https://openbiodiv.net/bab3438f-fdc8-4cc4-8cae-8461c9a07173

https://www.biodiversitylibrary.org/namedetail/Neophilaenus_campestris

https://www.catalogueoflife.org/data/taxon/46Q4H

https://treatment.plazi.org/id/7D5F7E45-B31C-D527-8263-159722370594

https://eol.org/pages/4215243

e7c8ed45-3cb5-4ca8-9bd8-a9c013beae7a

##### Notes

Distribution map (Fig. 6a) and habitus (Fig. 6b).

**Chresonymy**: *Ptyeluscampestris* Fall. in (Joakimov 1909)

**Literature data**: Pre-Balkan (Joakimov 1909)⁠; Sofia Plain (Joakimov 1909); Vitosha Mts. (Joakimov 1909, Bajrjamova 1984)⁠, Krupnik-Sandanski-Petrich Valley (Sander 1985)⁠; Southern Black Sea Coast (Sander 1985)⁠⁠.

**New data**: Danube Plain; Dobrudzha; Northern Black Sea Coast; Pre-Balkan; Western, Central and Eastern Stara Planina Mts.; Kraishtensko-Konjavo Region; Sofia Plain; Sub-Balkan valleys; Vitosha Mts.; Lozenska Planina Mts.; Sredna Gora Mts.; Ograzhden Mts.; Belasitsa Mts; Krupnik-Sandanski-Petrich Valley; Rila Mts.; Pirin Mts.; Slavjanka Mts.; Mesta River Valley; Rhodopes; Upper Thracian Lowland, Sakar-Tundzha Region; Strandzha Mts.; Southern Black Sea coast. Detailed occurrence data: Angelova and Gjonov (2024).

**Phenology**: April-November.

**Altitudinal distribution**: 0-1900 m a.s.l.

**Host plant**: Poaceae (Nickel 2003).

#### Neophilaenus exclamationis

(Thunberg, 1784)

0F11A4E0-D315-5404-B4F4-2340136A1F8C

https://www.gbif.org/species/4482797

http://www.boldsystems.org/index.php/Taxbrowser_Taxonpage?taxid=599324

https://openbiodiv.net/bab3438f-fdc8-4cc4-8cae-8461c9a07173

https://www.biodiversitylibrary.org/namedetail/Neophilaenus_exclamationis

https://www.catalogueoflife.org/data/taxon/46Q4J

Not available

https://eol.org/pages/4215241

77f4c877-b1c8-4ae6-81da-685741db5ebd

##### Notes

Distribution map (Fig. 7a) and habitus (Fig. 7b).

**Literature data**: Krupnik-Sandanski-Petrich Valley (Sander 1985); Belasitsa Mts. (Bajrjamova 1988⁠); Rila Mts. (Lindberg 1949)⁠; Rhodopes (Sander 1985, Gjonov 2004)⁠.

**New data**: Kraishtensko-Konjavo Region; Rhodopes; Upper Thracian Lowland. Detailed occurrence data: Angelova and Gjonov (2024).

**Phenology**: May-October.

**Altitudinal distribution**: 325-600 m a.s.l.

**Additional notes**: Rare in Bulgaria.

**Host plant**: Poaceae (*Festucaovina* L., *Deschampsiaflexuosa* (L.) Trin. and other grasses) (Nickel 2003).

#### Neophilaenus infumatus

(Haupt, 1917)

D340EA49-25DA-5BFD-B175-EFF509DF65DC

https://www.gbif.org/species/4482787

http://www.boldsystems.org/index.php/Taxbrowser_Taxonpage?taxid=1158184

https://www.biodiversitylibrary.org/namedetail/Neophilaenus_infumatus

https://www.catalogueoflife.org/data/taxon/46Q4K

Not available

https://eol.org/pages/4215248

5ed90469-f2ef-4e4d-bd59-181c0537c323

##### Notes

Distribution map (Fig. 8a) and habitus (Fig. 8b).

**Literature data**: Northern Black Sea coast (Pelov 1968); Sofia Plain (Bajrjamova 1970); Vitosha Mt. (Bajrjamova 1990)⁠.

**New data**: Western and Central Stara Planina Mts.; Kraishtensko-Konjavo Region; Vitosha Mts.; Belasitsa Mts; Rila Mts.; Pirin Mts.; Slavjanka Mts. Detailed occurrence data: Angelova and Gjonov (2024).

**Phenology**: May-October.

**Altitudinal distribution**: 600-2600 m a.s.l.

**Host plant**: *Festucaovina* group (Nickel 2003).

**Additional notes**: In Bulgaria, it occurs mainly in the high parts of the mountains.

#### Neophilaenus lineatus

(Linnaeus, 1758)

29770EF9-1F89-5C1F-94E6-970031D4F75E

https://www.gbif.org/species/4482791

http://www.boldsystems.org/index.php/Taxbrowser_Taxonpage?taxid=224076

https://openbiodiv.net/d41ee006-6da2-409b-a875-b460a206c17a

https://www.biodiversitylibrary.org/namedetail/Neophilaenus_lineatus

https://www.catalogueoflife.org/data/taxon/46Q4L

https://treatment.plazi.org/id/03BD1B1B-FFAF-FFFA-4DF0-FC4F8C3BEEEC

https://eol.org/pages/2869456

918f2704-0a72-4641-8c93-e664afb34bbd

##### Notes

Distribution map (Fig. 9a) and habitus (Fig. 9b).

**Chresonymy**: *Philaenuslineatus* Lin. in Nedjalkov (1908); *Ptyeluslineatus* L. in Joakimov (1909)

**Literature data**: Sofia Plain (Joakimov 1909)⁠; Rila Mts. (Joakimov 1909)⁠⁠; Vitosha Mts. (Nedjalkov 1908, Sander 1985)⁠; Krupnik-Sandanski-Petrich Valley (Sander 1985); Rhodopes (Sander 1985, Gjonov 2004); Sakar-Tundzhan Region (Joakimov 1909)⁠.

**New data**: Danube Plain; Pre-Balkan; Western, Central and Eastern Stara Planina Mts.; Kraishtensko-Konjavo Region; Sofia Plain; Vitosha Mts.; Lozenska Planina Mts.; Sredna Gora Mts.; Ograzhden Mts.; Belasitsa Mts; Krupnik-Sandanski-Petrich Valley; Rila Mts.; Pirin Mts.; Rhodopes; Strandzha Mts. Detailed occurrence data: Angelova and Gjonov (2024).

**Phenology**: May-October.

**Altitudinal distribution**: 0-1900 m a.s.l.

**Host plant**: Polyphagous on Poaceae, Cyperaceae, probably also Juncaceae and other families (Nickel 2003).

#### Neophilaenus minor

(Kirschbaum, 1868)

C77678BE-BF5D-55EE-A890-A73995CD4C03

https://www.gbif.org/species/4482777

http://www.boldsystems.org/index.php/Taxbrowser_Taxonpage?taxid=1062855

https://openbiodiv.net/de73828a-685a-4a4a-b7da-ced501d78c65

https://www.biodiversitylibrary.org/namedetail/Neophilaenus_minor

https://www.catalogueoflife.org/data/taxon/46Q4N

Not available

https://eol.org/pages/4215244

6b98517e-9e84-4b46-aa14-c4a8d722dc56

##### Notes

Distribution map (Fig. 10a) and habitus (Fig. 10b).

**Chresonymy**: *Ptyelusminor* Kb. in Joakimov (1909)

**Literature data**: Sofia Plain (Lindberg 1949)⁠; Vitosha Mts. (Joakimov 1909)⁠; Sredna Gora Mts. (Joakimov 1909); Krupnik-Sandanski-Petrich Valley (Sander 1985); Belasitsa Mts. (Bajrjamova 1988); Rhodopes (Sander 1985).

**New data**: Danube Plain; Dobrudzha; Pre-Balkan; Western Stara Planina Mts.; Kraishtensko-Konjavo Region; Vitosha Mts.; Ograzhden Mts.; Rila Mts.; Pirin Mts.; Slavjanka Mts.; Mesta River Valley; Rhodopes; Upper Thracian Lowland. Detailed occurrence data: Angelova and Gjonov (2024).

**Phenology**: March-September.

**Altitudinal distribution**: 150-2050 m a.s.l.

**Host plant**: *Corynephoruscanescens*, *Festucaovina*, *Koeleriaglauca* and probably species of fine-leaved grasses (Nickel 2003).

#### Philaenus

Stål, 1864

F102EC64-70B0-5BA1-8E37-C57E0E69B7CA

#### Philaenus signatus

Melichar, 1896

0F1F04E8-C80E-599D-B0FF-B5325207591D

https://www.gbif.org/species/2016065

http://www.boldsystems.org/index.php/Taxbrowser_Taxonpage?taxid=198954

https://openbiodiv.net/b3d1e94c-5f18-4970-97df-4b04b1cd4e7c

https://www.biodiversitylibrary.org/namedetail/Philaenus_signatus

https://www.catalogueoflife.org/data/taxon/4G4SJ

Not available

https://eol.org/pages/858360

f11ca065-ef98-40ff-9928-caba45ccab98

##### Notes

Distribution map (Fig. 11a) and habitus (Fig. 11b).

**Literature data**: Sofia Plain (Bajrjamova 1990); Vitosha Mts. (Nedjalkov 1908)⁠. Both records are uncertain as the recorded locations do not match the ecological requirements of the species.

**New data**: Mesta River Valley; Southern Black Sea coast. Detailed occurrence data: Angelova and Gjonov (2024).

**Phenology**: May-August.

**Altitudinal distribution**: 0-550 m a.s.l.

**Host plant**: In Greece, adults of the species have been collected on various shrubs and trees, especially those of the genera *Arbutus* and *Quercus* (Drosopoulos and Asche 2000). As of now, the only recorded plant of the nymph and the freshly emerged adults is *Asphodelusmicrocarpus* Parl (Asphodelaceae) (Drosopoulos 2003). In Bulgaria, during the early summer, it was collected on a new host plant of *Asphodelinelutea* (L.) Rchb. (Asphodelaceae). Later in the season, when the plant's ground mass had wilted, it moved as adults on to woody species of the genus *Quercus*.

**Additional notes**: In Greece, it usually lives in the same habitat as *Philaenusspumarius*, more often near the sea, but there are also isolated records from the mountains (Drosopoulos and Asche 2000). In Bulgaria, it has been collected along the southern Black Sea coast and there are also isolated records in the mountains, near the border with Greece. Colour polymorphism is observed. Rare in Bulgaria.

#### Philaenus spumarius

(Linnaeus, 1758)

35849602-54D6-54CF-9195-AE73039FF669

https://www.gbif.org/species/2016038

http://www.boldsystems.org/index.php/Taxbrowser_Taxonpage?taxid=25797

https://openbiodiv.net/86e0a328-4765-48b7-95b2-197f34d82e76

https://www.biodiversitylibrary.org/namedetail/Philaenus_spumarius

https://www.catalogueoflife.org/data/taxon/4G4SL

https://treatment.plazi.org/id/7D5F7E45-B31C-D527-8263-159722370594

https://eol.org/pages/1691692

a8afb1c7-f6c9-4c90-97ca-e013e0b55dbf

##### Notes

Distribution map (Fig. 12a) and habitus (Fig. 12b).

**Chresonymy**: *Ptyelusspumarius* L. in Joakimov (1909)

**Literature data**: Danube Plain (Dirimanov and Harizanov 1964, Bairamova 1978, Angelova et al. 2008)⁠; Northern Black Sea Coast (Nedjalkov 1908, Bayryamova 1976, Bairamova 1978, Sander 1985); Pre-Balkan (Nedjalkov 1908, Pelov 1968, Bairamova 1978); Western Stara Planina (Bairamova 1978); Central Stara Planina Mts. ⁠(Cantoreanu and Gruev 1967); Sofia Plain (Bajrjamova 1970, Bayryamova 1976)⁠; Vitosha Mts. (Joakimov 1909, Lindberg 1949, Cantoreanu and Gruev 1967, Bairamova 1978)⁠⁠⁠; Lozen Mts. (Bayryamova 1976, Bairamova 1978); Plana Mts. (Bairamova 1978); Sub-Balkan valley (Dirimanov and Harizanov 1964, Cantoreanu and Gruev 1967, Bairamova 1978)⁠; Sredna Gora Mts. (Joakimov 1909)⁠; Krupnik-Sandanski-Petrich Valley (Sander 1985); Belasitsa Mts. (Bajrjamova 1988); Rila Mts. (Joakimov 1909, Lindberg 1949)⁠; Rhodopes (Dirimanov and Harizanov 1964, Sander 1985, Gjonov 2004); Sakar-Tundzha Region (Joakimov 1909)⁠⁠; Strandzha Mts. (Bayryamova 1976, Bairamova 1978).

**New data**: Danube Plain; Dobrudzha; Northern Black Sea Coast; Pre-Balkan; Western, Central and Eastern Stara Planina Mts.; Kraishtensko-Konjavo Region; Sofia Plain; Sub-Balkan valleys; Vitosha Mts.; Lozenska Planina Mts.; Sredna Gora Mts.; Ograzhden Mts.; Belasitsa Mts.; ⁠Krupnik-Sandanski-Petrich Valley; Rila Mts.; Pirin Mts.; Slavjanka Mts.; Mesta River Valley; Rhodopes; Upper Thracian Lowland; Sakar-Tundzha Region; Strandzha Mts.; Southern Black Sea coast. Detailed occurrence data: Angelova and Gjonov (2024).

**Phenology**: May-November.

**Altitudinal distribution**: 0-2200 m a.s.l.

**Host plant**: Extremely polyphagous (Nickel 2003, Thompson et al. 2023).

**Additional notes**: Eurytopic. Colour polymorphism is observed (Nickel 2003). Common in Bulgaria.

#### Cercopidae

Leach, 1815

795C9AA6-A875-5AF0-9BCC-052660AE3F42

#### Cercopis

Fabricius, 1775

3E1DC97D-DD80-5985-9EB5-4B3BE2E16F91

#### Cercopis arcuata

Fieber, 1844

82A18C6A-D4D9-5DC6-8CFE-06097384449F

https://www.gbif.org/species/2018563

http://www.boldsystems.org/index.php/Taxbrowser_Taxonpage?taxid=957182

https://openbiodiv.net/eefe22b5-dbb4-484b-a7ae-52ac1aa95736

https://www.biodiversitylibrary.org/namedetail/Cercopis_arcuata

https://www.catalogueoflife.org/data/taxon/ST4W

Not available

https://eol.org/pages/1079862

acff53f2-02a6-410a-b12a-27905eca4610

##### Notes

Distribution map (Fig. 13a) and habitus (Fig. 13b).

**Chresonymy**: *Triecphoraarcuata* Fieb. in Nedjalkov (1908)

**Literature data**: Rila Mts. (Sander 1985)⁠.

**New data**: Pre-Balkan; Western, Central and Eastern Stara Planina Mts.; Kraishtensko-Konjavo Region; Sofia Plain; Vitosha Mts.; Lozenska Planina Mts.; Sredna Gora Mts.; Ograzhden Mts.; Belasitsa Mts.; ⁠Krupnik-Sandanski-Petrich Valley; Rila Mts.; Pirin Mts.; Rhodopes; Sredna Gora Mts.; Upper Thracian Lowland; Sakar-Tundzha Region; Strandzha Mts.; Southern Black Sea coast. Detailed occurrence data: Angelova and Gjonov (2024).

**Phenology**: April-November.

**Altitudinal distribution**: 0-1800 m a.s.l.

**Host plant**: Pine or oak forests, with the majority of adults discovered amongst dicotyledonous herbs in the Czech Republic, Austria and France (Nickel 2003).

#### Cercopis intermedia

Kirschbaum, 1868

5AA39074-F3A6-5D3C-ABD0-3BA941475024

https://www.gbif.org/species/2019168

http://www.boldsystems.org/index.php/Taxbrowser_Taxonpage?taxid=508392

https://openbiodiv.net/07f22db6-56c1-40a4-85e2-8454f895e3dd

https://www.biodiversitylibrary.org/namedetail/Cercopis_intermedia

https://www.catalogueoflife.org/data/taxon/ST8Q

Not available

https://www.catalogueoflife.org/data/taxon/ST8Q

48a284f7-8f3d-4501-b85b-18687594dfe3

##### Notes

Distribution map (Fig. 14a) and habitus (Fig. 14b).

**Literature data**: Danube Plain (Cantoreanu and Gruev 1967)⁠; Northern Black Sea coast (Sander 1985); Sub-Balkan valleys (Dirimanov and Harizanov 1964); Sredna Gora Mts. (Dirimanov and Harizanov 1964)⁠; Rhodopes (Gjonov 2004); Upper Thracian Lowland (Dirimanov and Harizanov 1964)⁠⁠.

**New data**: Danube Plain; Dobrudzha; Northern Black Sea Coast; Pre-Balkan; Sofia Plain; Sredna Gora Mts.; Rhodopes; Upper Thracian Lowland; Sakar-Tundzha Region; Strandzha Mts.; Southern Black Sea coast. Detailed occurrence data: Angelova and Gjonov (2024).

**Phenology**: April-June.

**Altitudinal distribution**: 0-1000 m a.s.l.

**Host plant**: On plants like *Astragalus* L., *Onopordum* L., *Verbascum* L. and *Medicagosativa* L. and trees, such as *Pistaciavera* L., *Prunusdomestica* L., *Acacia* spp., *Salix* spp. and *Alnus* spp. (Lodos and Kalkandelen 1981).

**Additional notes**: Common in karst areas.

#### Cercopis sanguinolenta

(Scopoli, 1763)

F9B98FD7-5B8E-52CE-8E67-1955A86423F5

https://www.gbif.org/species/2018539

http://www.boldsystems.org/index.php/Taxbrowser_Taxonpage?taxid=463458

https://openbiodiv.net/7c02203f-9246-4c4f-bd56-9ad8b1abcdeb

https://www.biodiversitylibrary.org/namedetail/Cercopis_sanguinolenta

https://www.catalogueoflife.org/data/taxon/STBM

Not available

https://eol.org/pages/1080480

4028282a-bed4-44e3-8a49-ade5fec62ec7

##### Notes

Distribution map (Fig. 15a) and habitus (Fig. 15b).

**Chresonymy**: *Triecphoramactata* Germ. in Nedjalkov (1908); *Triecphorasanguinolenta* Lin. in Nedjalkov (1908); *Triecphoramactata* Germ. in Joakimov (1909); *Cercopissanguinea* Geoffr. in Lindberg (1949); *Cercopissanguinea* Geoffr. 1785 in Cantoreanu and Gruev (1967).

**Literature data**: Central Stara Planina Mts. (Cantoreanu and Gruev 1967); Sofia Plain (Joakimov 1909); Kraishtensko-Konjavo Region (Joakimov 1909); Vitosha Mts. (Joakimov 1909, Bajrjamova 1990)⁠; Ljulin Mts. (Joakimov 1909); Sredna Gora Mts. (Dirimanov and Harizanov 1964); Krupnik-Sandanski-Petrich Valley (Joakimov 1909)⁠; Belasitsa Mts. (Bajrjamova 1988); Rila Mts. (Lindberg 1949); Rhodopes (Cantoreanu and Gruev 1967, Gjonov 2004)⁠; Upper Thracian Lowland (Nedjalkov 1908, Dirimanov and Harizanov 1964); Southern Black Sea Coast (Bayryamova 1976).

**New data**: Danube Plain; Northern Black Sea Coast; Pre-Balkan; Western, Central and Eastern Stara Planina Mts.; Kraishtensko-Konjavo Region; Sofia Plain; Vitosha Mts.; Lozenska Planina Mts.; Ograzhden Mts.; Belasitsa Mts.; ⁠Krupnik-Sandanski-Petrich Valley; Rila Mts.; Pirin Mts.; Slavjanka Mts.; Mesta River Valley; Rhodopes; Sakar-Tundzha Region; Strandzha Mts.; Southern Black Sea coast. Detailed occurrence data: Angelova and Gjonov (2024).

**Phenology**: March-August.

**Altitudinal distribution**: 0-1800 m a.s.l.

**Host plant**: Polyphagous on various grasses and herbs (Nickel 2003).

#### Cercopis vulnerata

(Rossi, 1807)

9063888E-68E5-5C33-99EC-55E255768A80

https://www.gbif.org/species/2018510

http://www.boldsystems.org/index.php/Taxbrowser_Taxonpage?taxid=348682

https://openbiodiv.net/47ec813b-8d95-40db-bf27-620e558f2bb0

https://www.biodiversitylibrary.org/namedetail/Cercopis_vulnerata

https://www.catalogueoflife.org/data/taxon/5XKBY

Not available

https://eol.org/pages/1080492

df53c592-016a-4177-9b03-044e984ea1da

##### Notes

Distribution map (Fig. 16a) and habitus (Fig. 16b).

**Chresonymy**: *Triecphoravulnerata* Illig. in Joakimov (1909)

**Literature data**: Sredna Gora Mts. (Joakimov 1909); Krupnik-Sandanski-Petrich Valley (Sander 1985).

**New data**: Western Stara planina Mts.; Rila Mts.; Strandzha Mts.; Southern Black Sea coast. Detailed occurrence data: Angelova and Gjonov (2024).

**Phenology**: April-July.

**Altitudinal distribution**: 0-1700 m a.s.l.

**Host plant**: Adults polyphagous on various tall herbs and grasses, Nymphs subterranean on Poaceae roots (Nickel 2003).

**Additional notes**: Rare in Bulgaria.

## Discussion

Bulgaria has a relatively rich species diversity of the superfamily Cercopoidea in Europe due to the Bulgarian geographical location and varied topography, which provide conditions for different habitats. Out of 38 species known for Europe, 16 species have been recorded for the country and one species, *Haematolomadorsatum*, is in need of confirmation. The majority of species in Bulgaria have a wide distribution and can be found at various altitudes. However, certain species, such as *Aphrophoracorticea*, *Neophilaenusexclamationis* and *Cercopisvulnerata*, are only found in a few localities and the distribution of *Neophilaenusinfumatus* is restricted to the high mountains of Bulgaria.

The species present in Bulgaria are classified according to the classification of Vigna Taglianti et al. (1999) in the following faunal complexes: Holarctic - 3 species (*L.coleoptrata*, *N.lineatus* and *Ph.spumarius*), Palaearctic - 2 species (*A.alni* and *A.salicina*), Central-asiatic-European - 3 species (*A.corticea*, *N.albipennis* and *N.infumatus*), Turano-European - 2 species (*N.minor* and *C.sanguinolenta*), Turano-Mediterranean - 2 species (*Ph.signatus* and *C.intermedia*), Europeo-Mediterranean - 3 species (*N.campestris*, *N.exclamationis* and *C.vulnerata*) and one European species (*C.arcuata*).

Most species are broad oligophagous or polyphagous, with the exception of *Aphrophoracorticea*, which is monophagous on pines and *Philaenussignatus*, whose nymphs and freshly-emerged adults are known to be monophagous on *Asphodelusmicrocarpus*. A new food plant, *Asphodelinelutea*, has been established for the species in Bulgaria in the current study.

The 25-year study of Cercopoidea in Bulgaria has yielded 8722 digitised specimens from 888 locations. These data could be a valuable reference for future research and monitoring.

## Supplementary Material

XML Treatment for Aphrophoridae

XML Treatment for Aphrophora

XML Treatment for Aphrophora alni

XML Treatment for Aphrophora corticea

XML Treatment for Aphrophora salicina

XML Treatment for Lepyronia

XML Treatment for Lepyronia coleoptrata

XML Treatment for Neophilaenus

XML Treatment for Neophilaenus albipennis

XML Treatment for Neophilaenus campestris

XML Treatment for Neophilaenus exclamationis

XML Treatment for Neophilaenus infumatus

XML Treatment for Neophilaenus lineatus

XML Treatment for Neophilaenus minor

XML Treatment for Philaenus

XML Treatment for Philaenus signatus

XML Treatment for Philaenus spumarius

XML Treatment for Cercopidae

XML Treatment for Cercopis

XML Treatment for Cercopis arcuata

XML Treatment for Cercopis intermedia

XML Treatment for Cercopis sanguinolenta

XML Treatment for Cercopis vulnerata

## Figures and Tables

**Table:** 

Nanopublication	Creator	Date

**Figure 1a.**
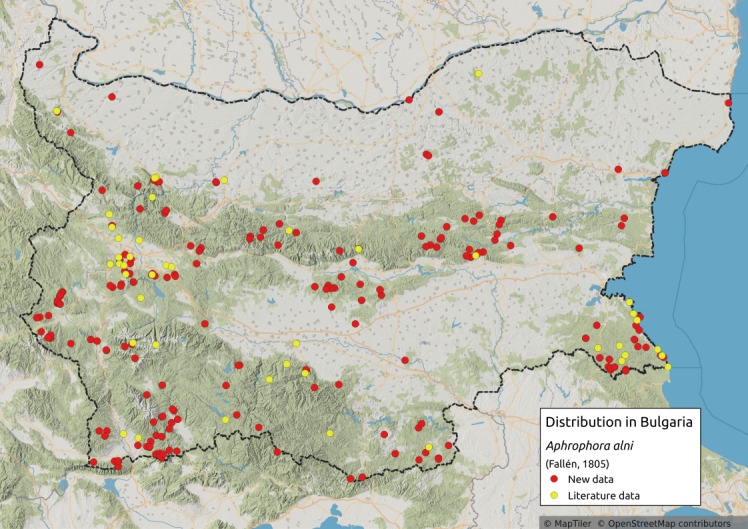
Distribution map in Bulgaria;

**Figure 1b.**
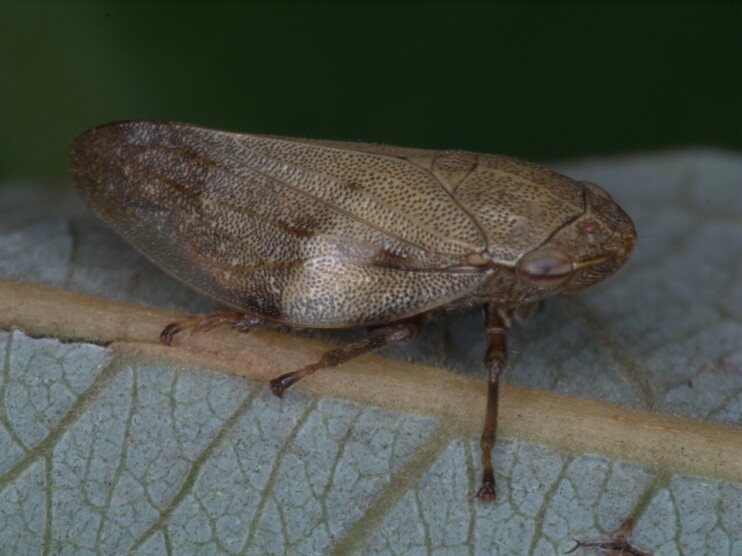
Habitus (photo: I. Gjonov).

**Figure 2a.**
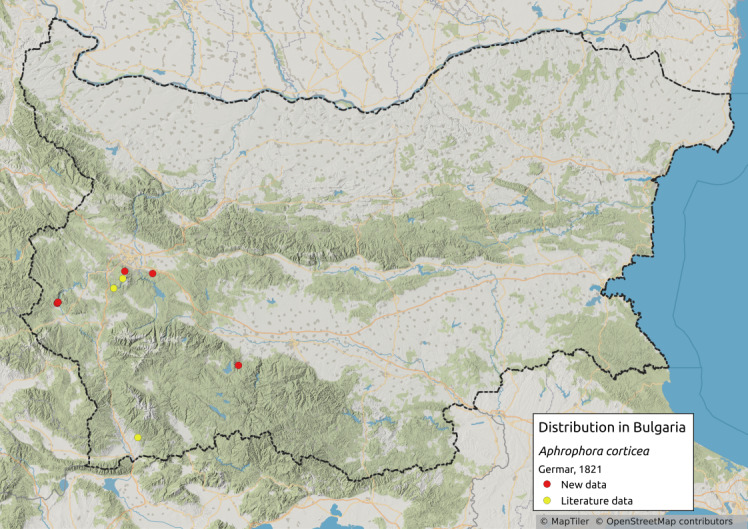
Distribution map in Bulgaria;

**Figure 2b.**
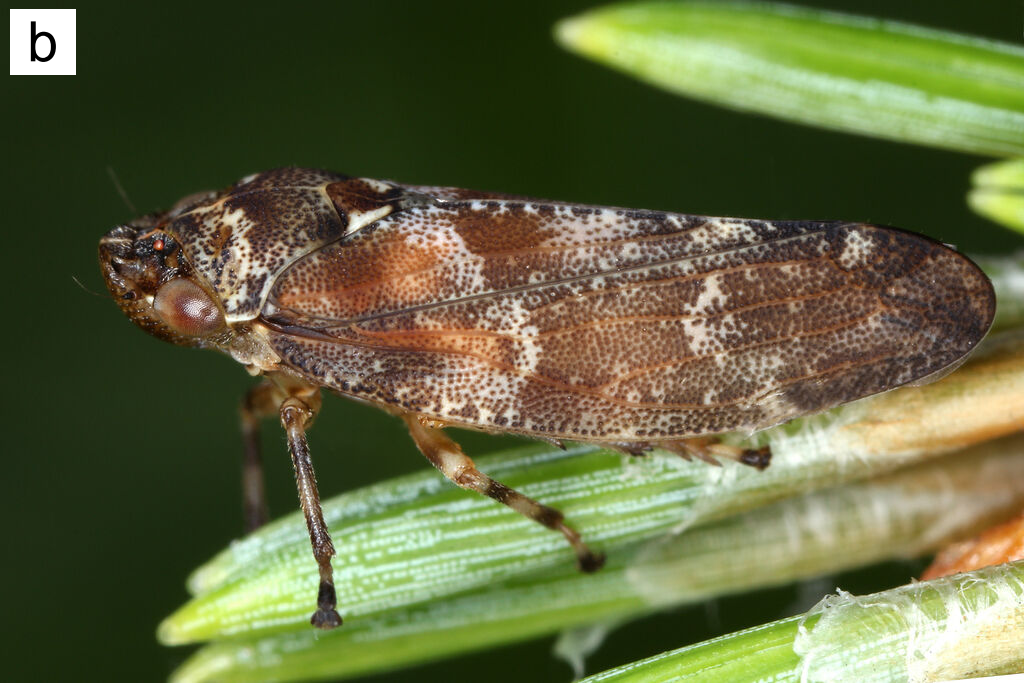
Habitus, specimen from Germany (photo: G. Kunz, https://www.inaturalist.org/observations/66533826).

**Figure 3a.**
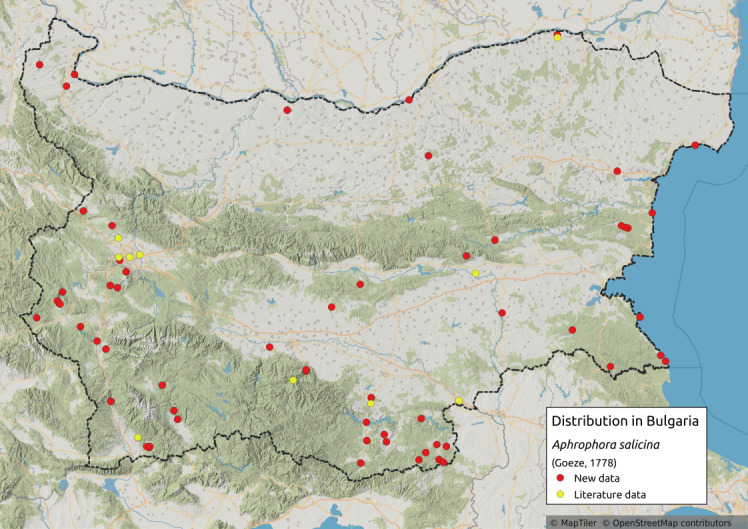
Distribution map in Bulgaria;

**Figure 3b.**
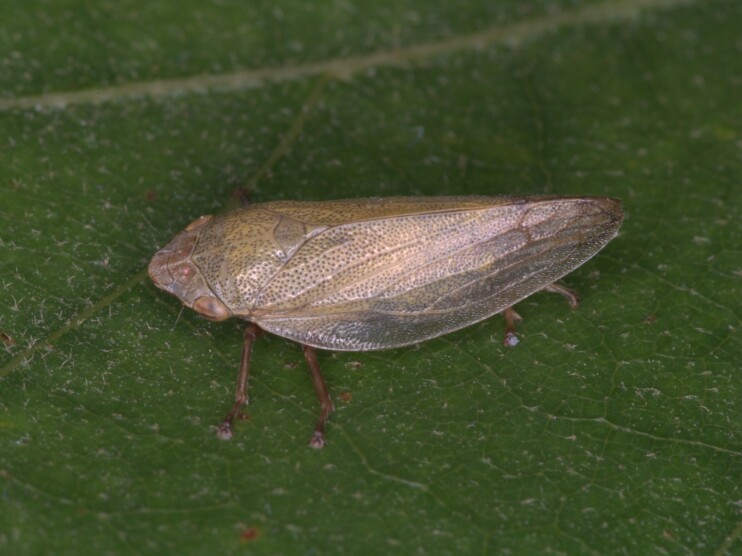
Habitus (photo: I. Gjonov).

**Figure 4a.**
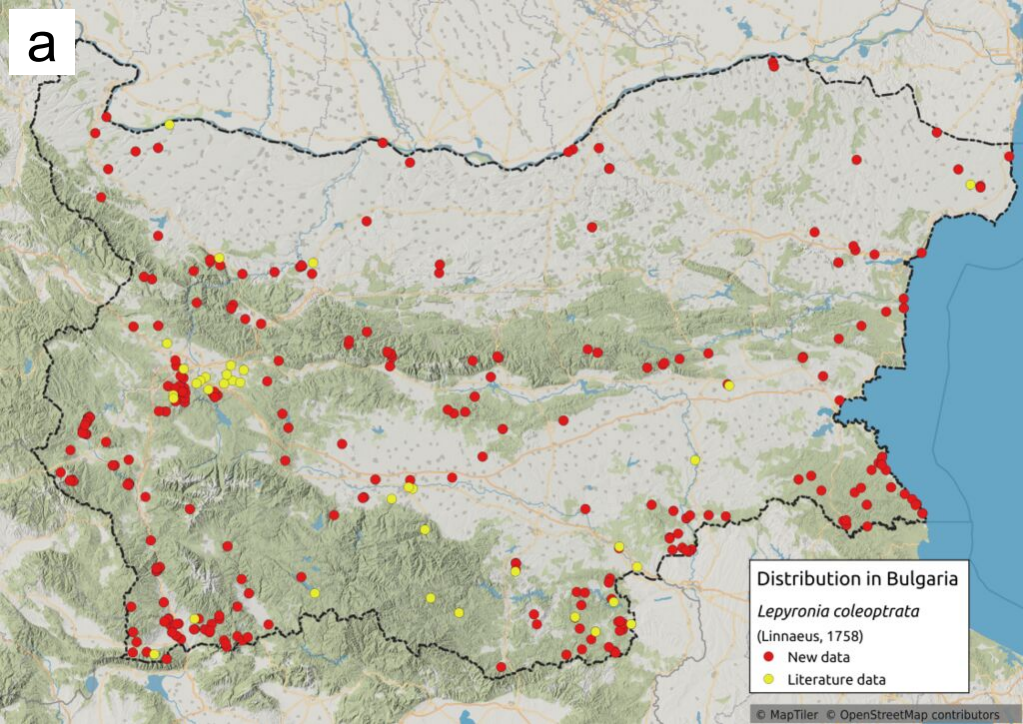
Distribution map in Bulgaria;

**Figure 4b.**
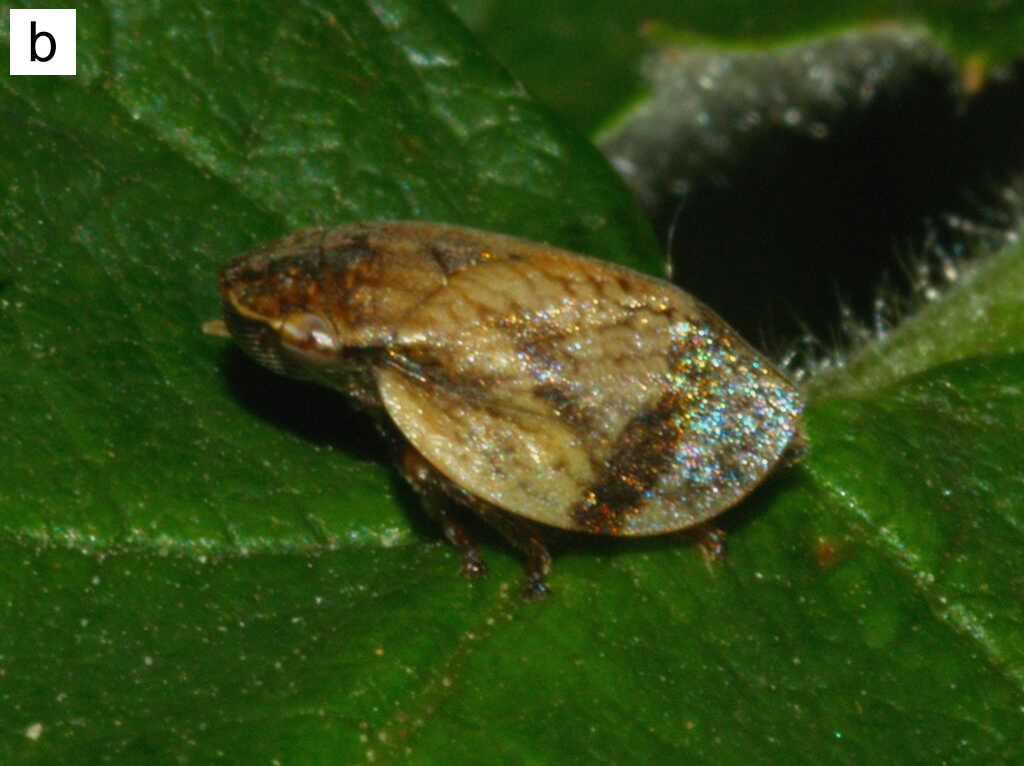
Habitus (photo: I. Gjonov).

**Figure 5a.**
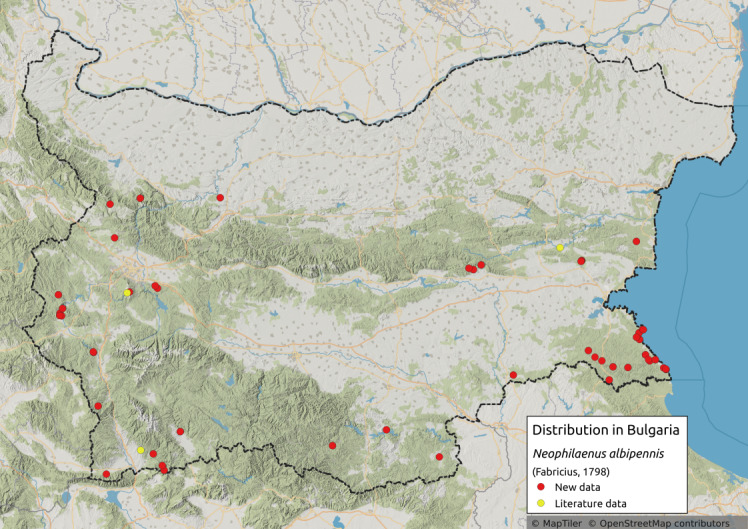
Distribution map in Bulgaria;

**Figure 5b.**
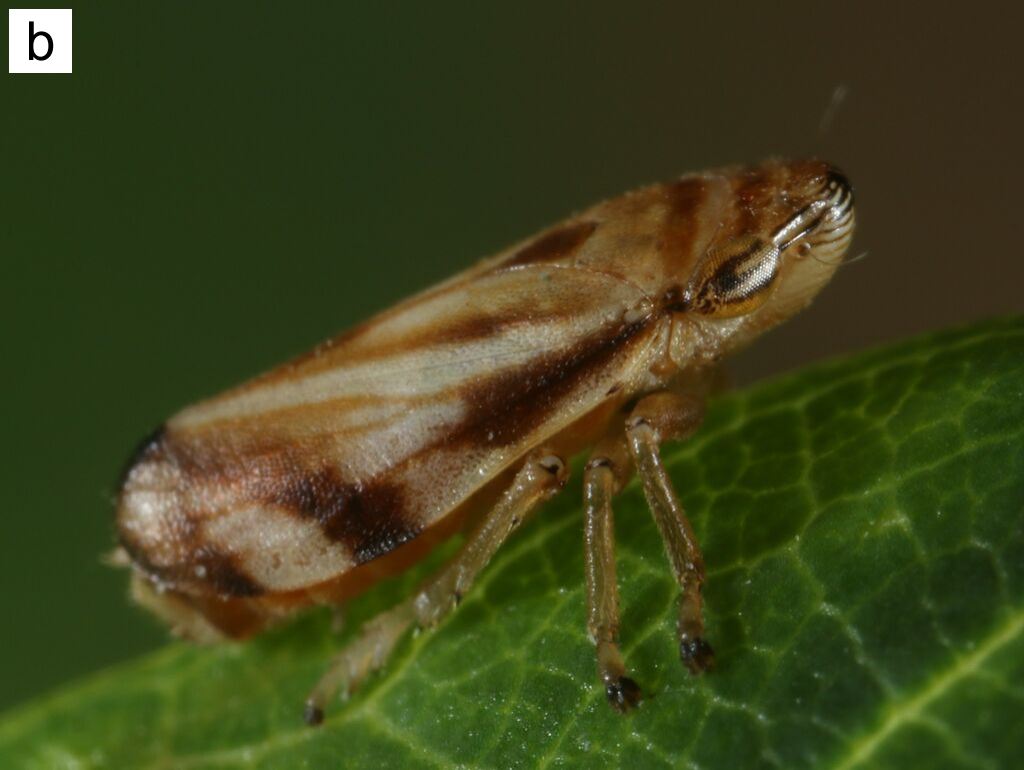
Habitus (photo: I. Gjonov).

**Figure 6a.**
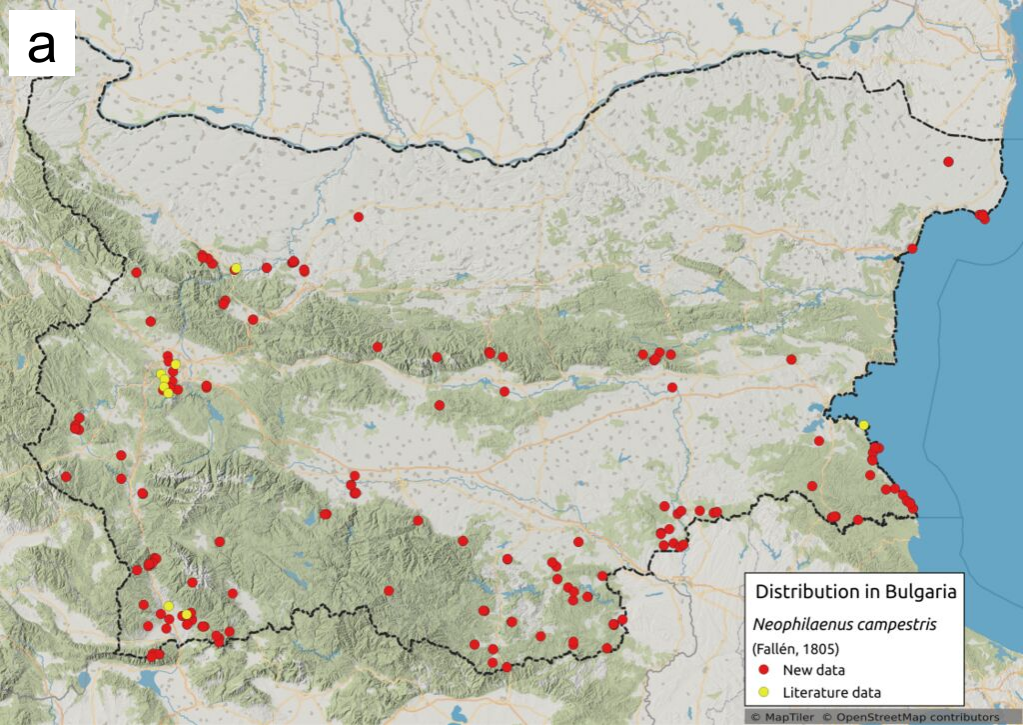
Distribution map in Bulgaria;

**Figure 6b.**
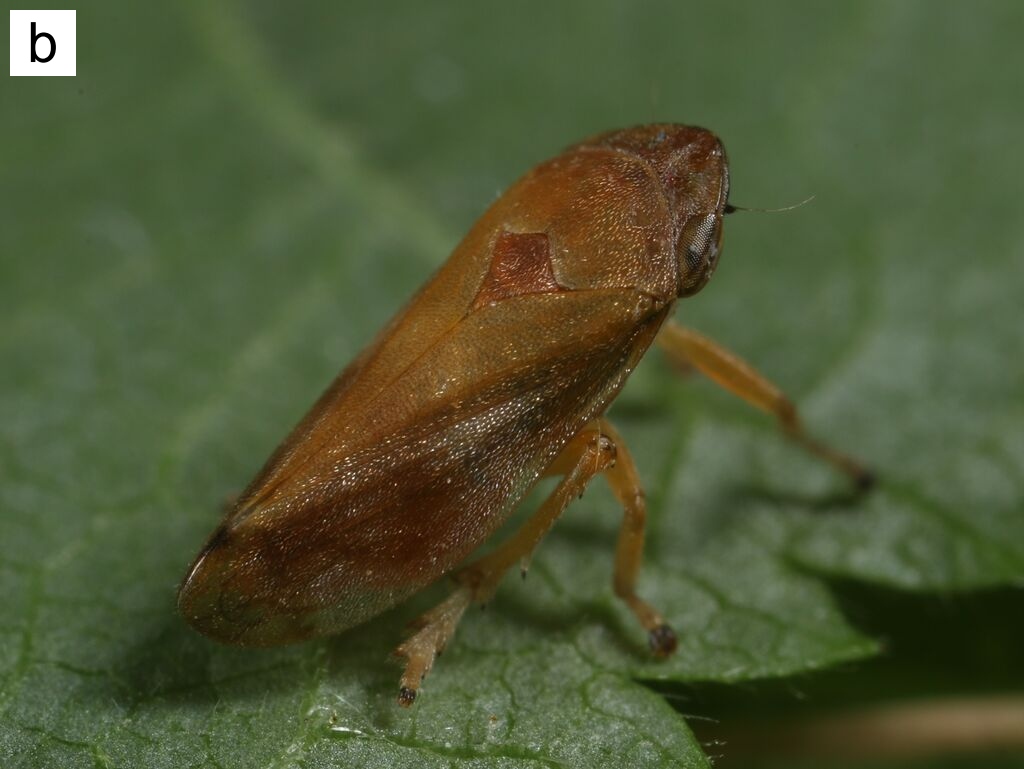
Habitus (photo: I. Gjonov).

**Figure 7a.**
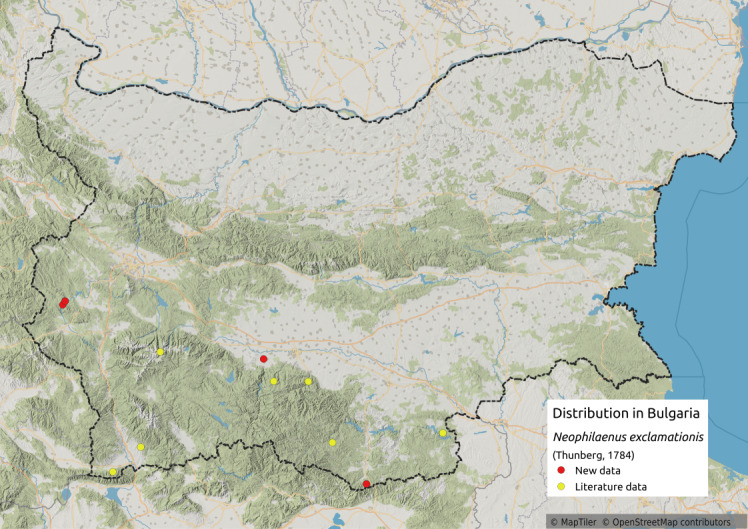
Distribution map in Bulgaria;

**Figure 7b.**
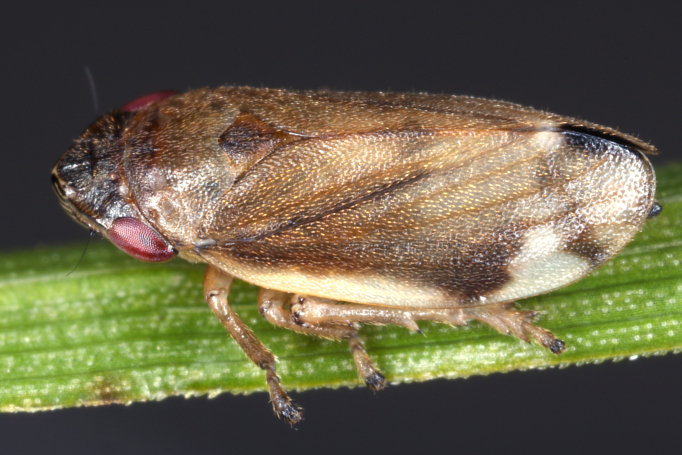
Habitus, specimen from Austria (photo: G. Kunz, https://www.inaturalist.org/observations/51116874).

**Figure 8a.**
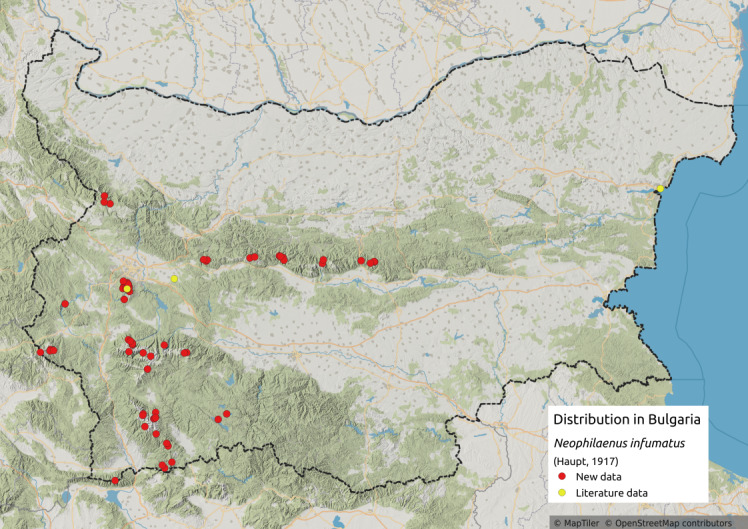
Distribution map in Bulgaria;

**Figure 8b.**
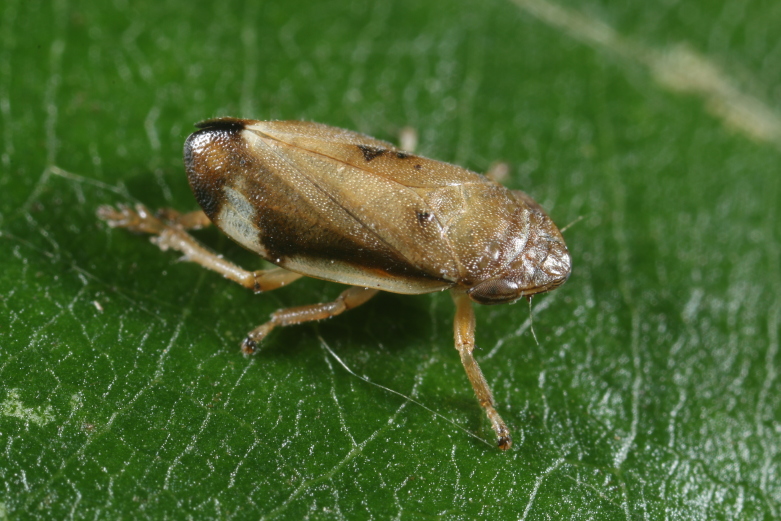
Habitus (photo: I. Gjonov).

**Figure 9a.**
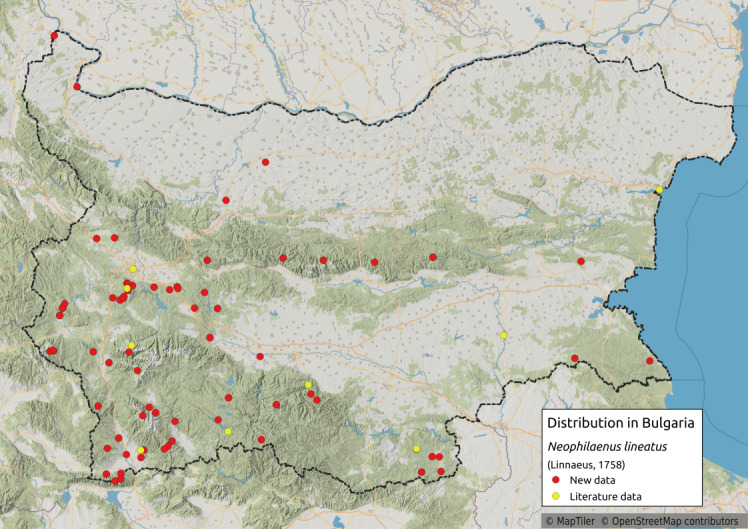
Distribution map in Bulgaria;

**Figure 9b.**
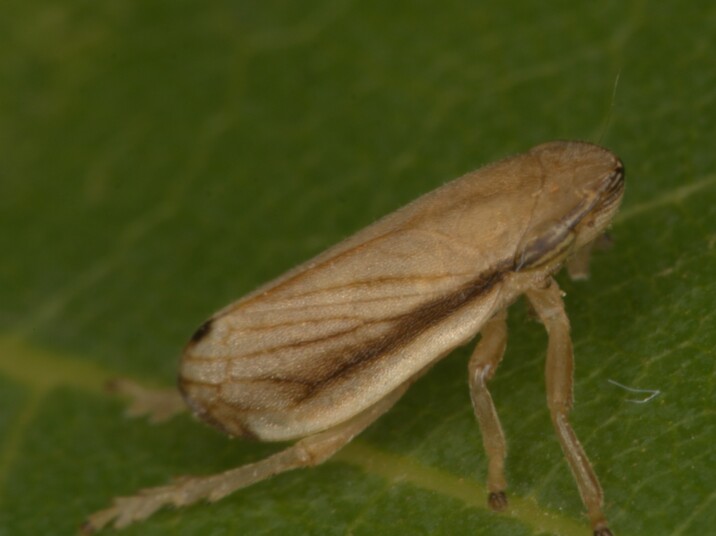
Habitus (photo: I. Gjonov).

**Figure 10a.**
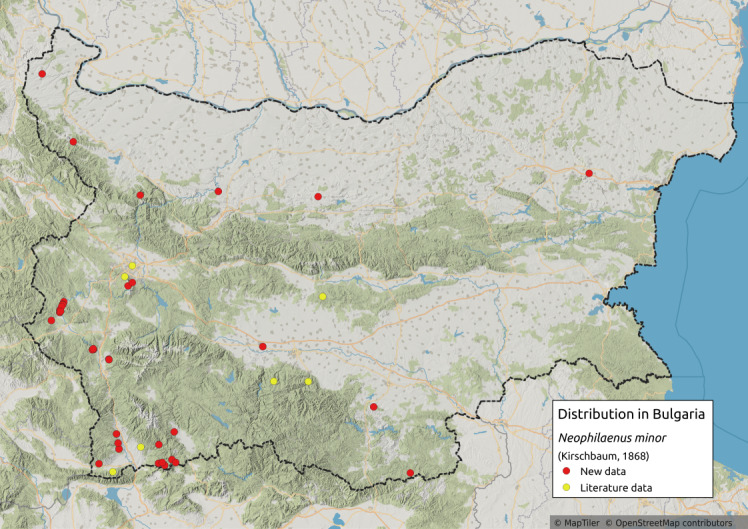
Distribution map in Bulgaria;

**Figure 10b.**
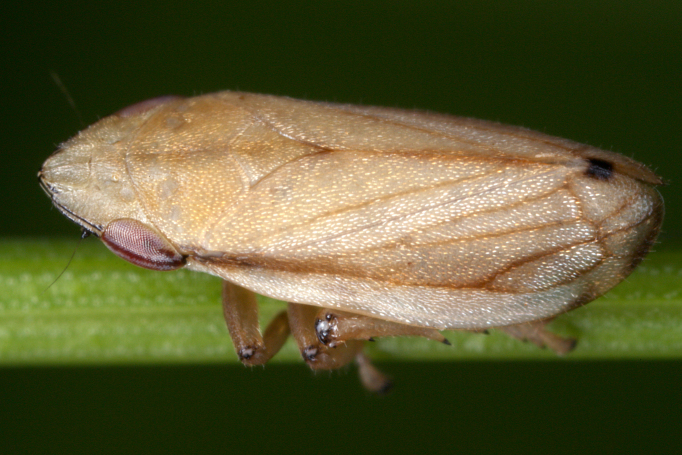
Habitus, specimen from Croatia (photo: G. Kunz, https://www.inaturalist.org/observations/66521555).

**Figure 11a.**
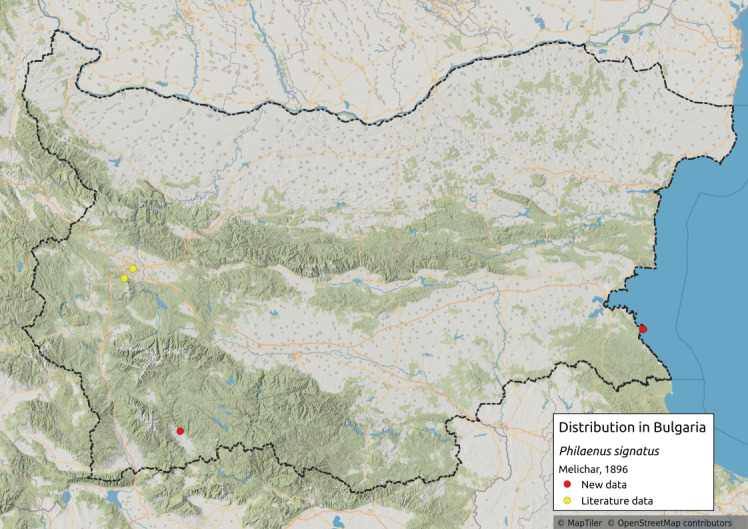
Distribution map in Bulgaria;

**Figure 11b.**
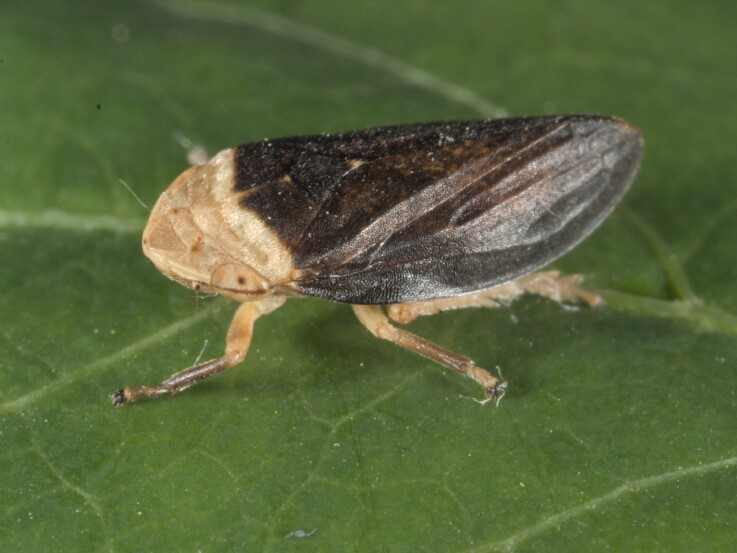
Habitus - one of several colour forms (photo: I. Gjonov).

**Figure 12a.**
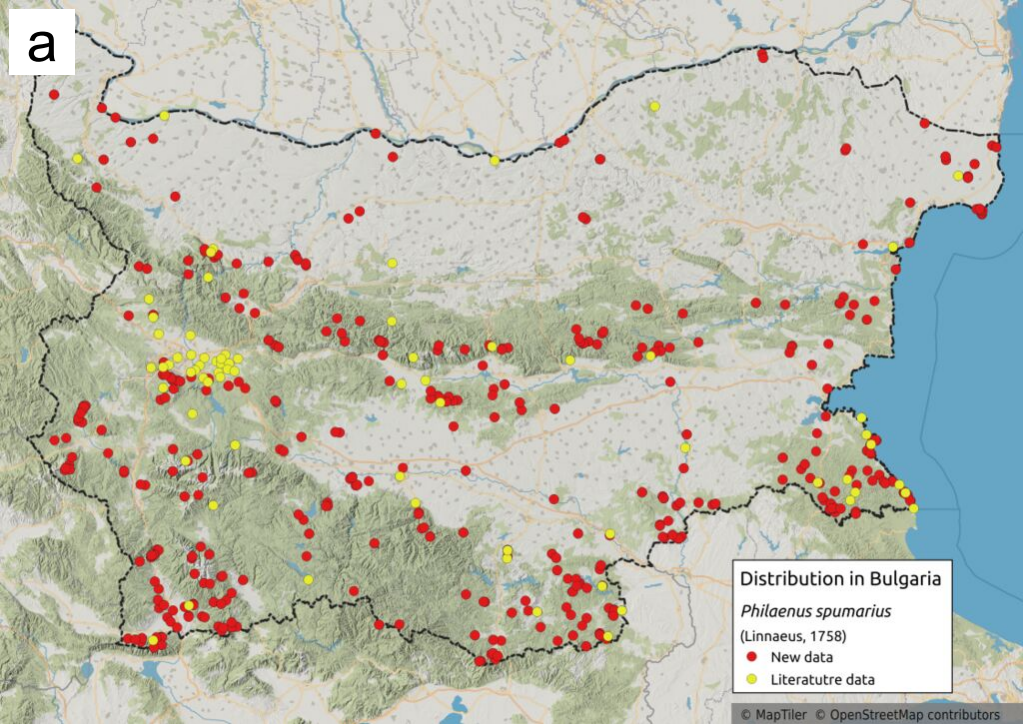
Distribution map in Bulgaria;

**Figure 12b.**
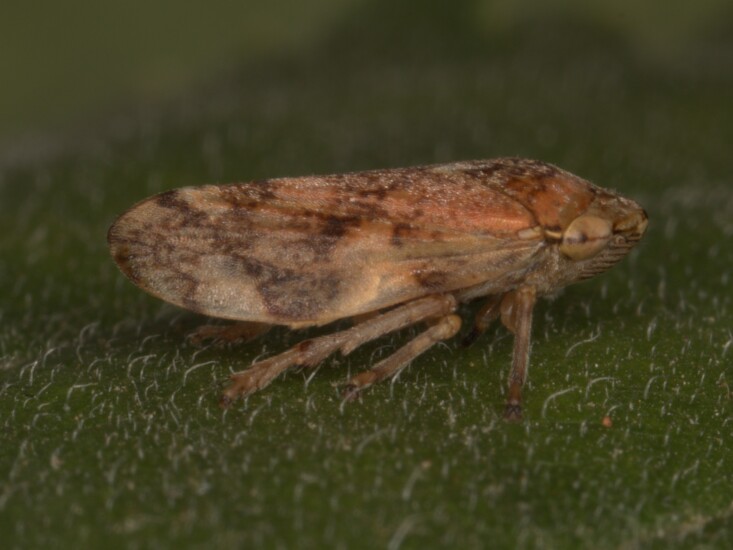
Habitus - the most common of several colour forms (photo: I. Gjonov).

**Figure 13a.**
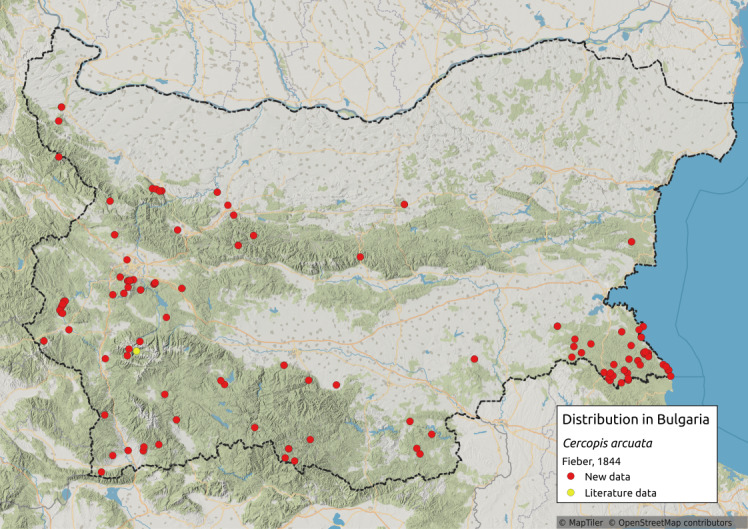
Distribution map in Bulgaria;

**Figure 13b.**
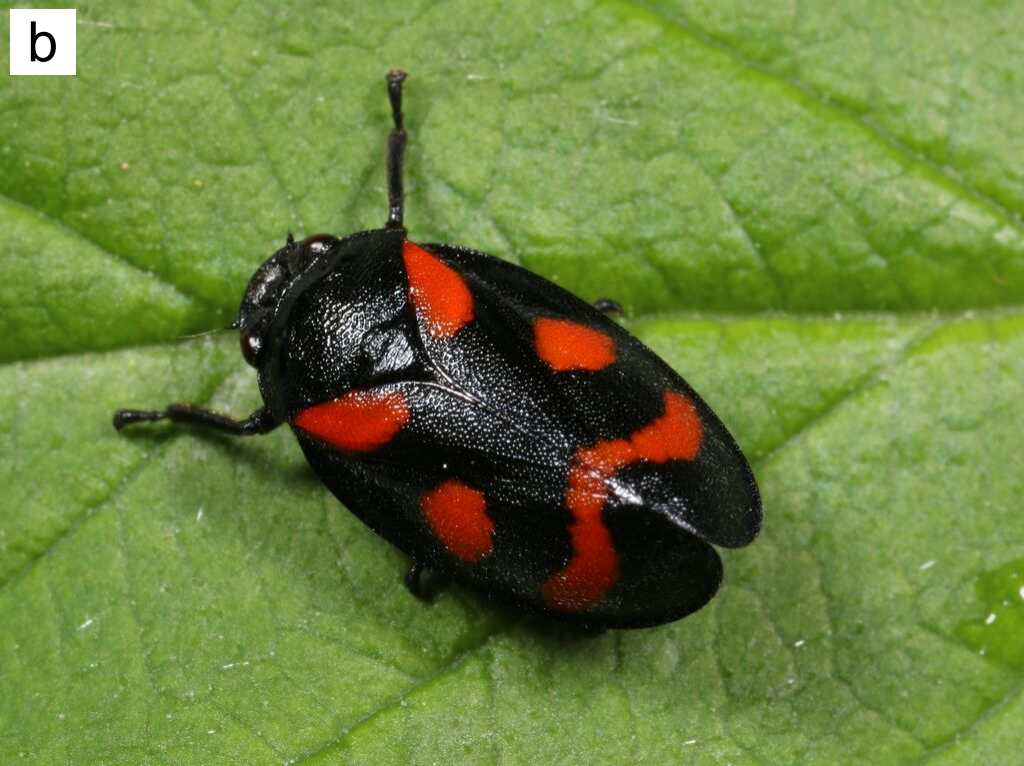
Habitus (photo: I. Gjonov).

**Figure 14a.**
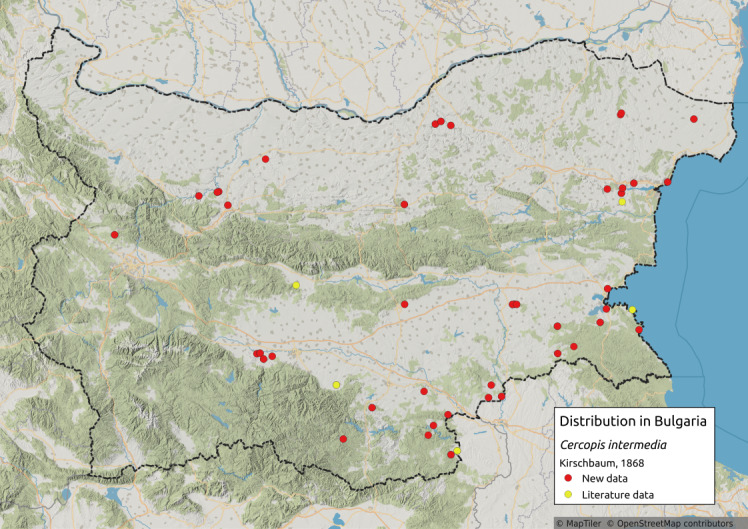
Distribution map in Bulgaria;

**Figure 14b.**
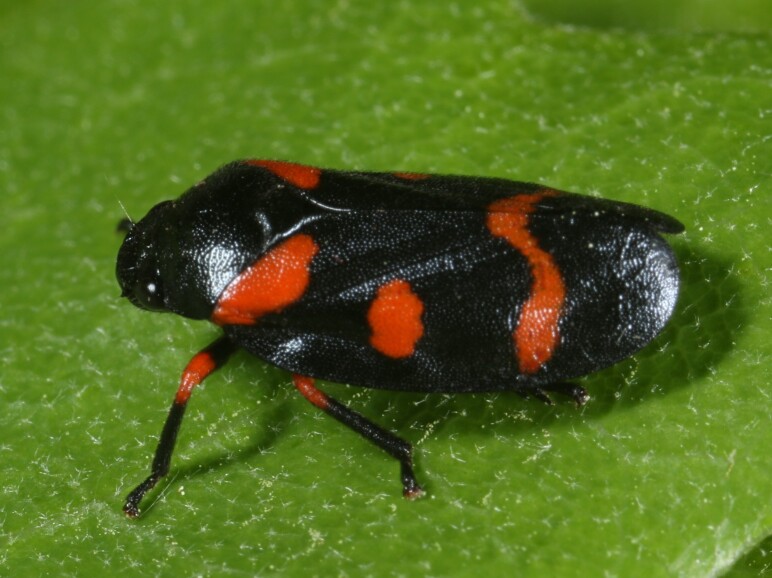
Habitus (photo: I. Gjonov).

**Figure 15a.**
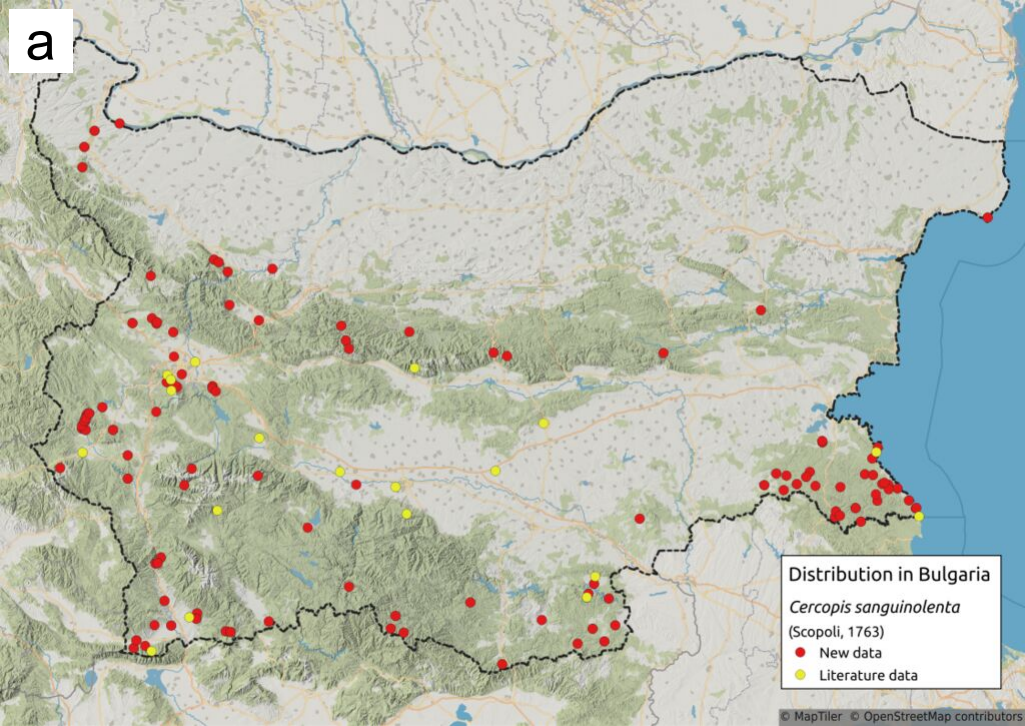
Distribution map in Bulgaria;

**Figure 15b.**
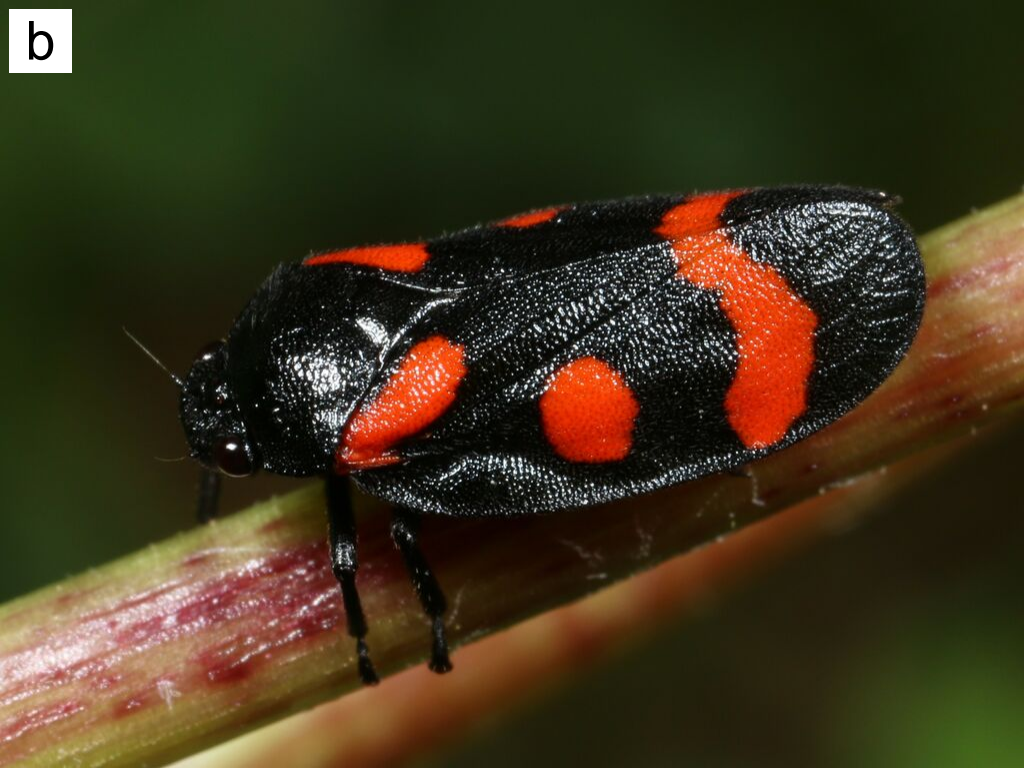
Habitus (photo: I. Gjonov).

**Figure 16a.**
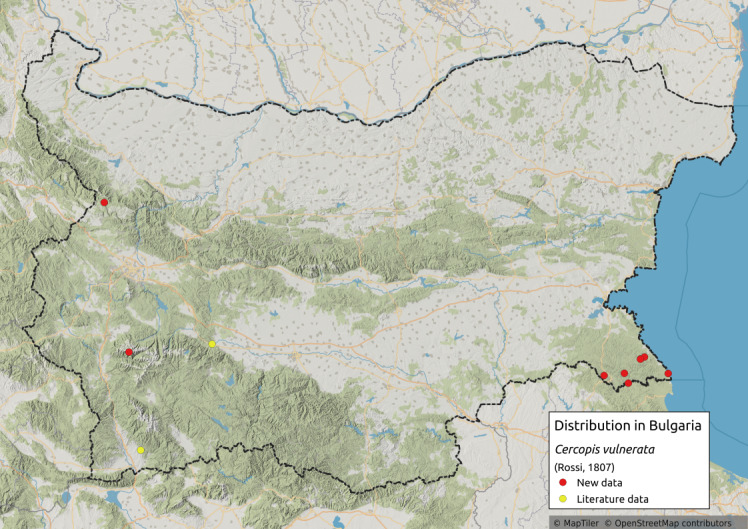
Distribution map in Bulgaria;

**Figure 16b.**
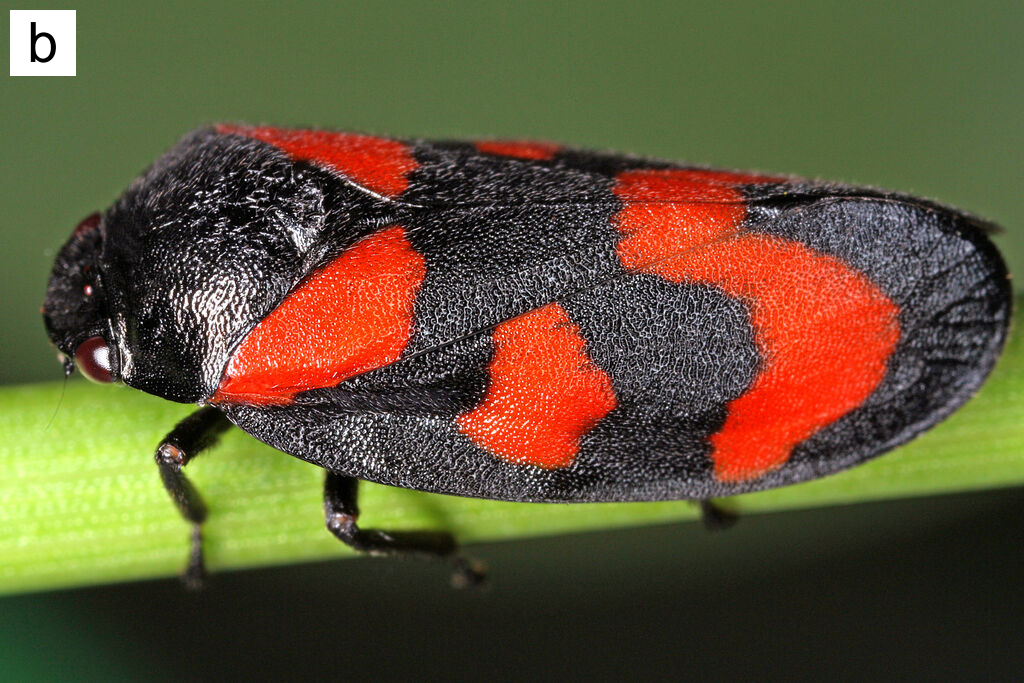
Habitus, specimen from Austria (photo: G. Kunz, https://www.inaturalist.org/observations/66312129).
